# Narcissism and Social Media: The Role of Communal Narcissism

**DOI:** 10.3390/ijerph181910106

**Published:** 2021-09-26

**Authors:** Kolbrun Harpa Kristinsdottir, Haukur Freyr Gylfason, Rannveig Sigurvinsdottir

**Affiliations:** 1 Department of Psychology, Reykjavik University, 102 Reykjavik, Iceland; kolbrunk16@ru.is (K.H.K.); rannveigssig@gmail.com (R.S.); 2 Department of Business, Reykjavik University, 102 Reykjavik, Iceland

**Keywords:** personality, communal narcissism, social networking sites, social media

## Abstract

Agentic narcissism and vulnerable narcissism have been widely studied in relation to social media use. However, with research on communal narcissism in its early stages, the current study examines communal narcissism in relation to social media use. Specifically, the current study investigates whether communal narcissism is related to use and frequency of use of the popular social networking sites Instagram, Reddit and Twitter, and if communal narcissism relates to the importance of receiving feedback and to the quality-rating of self-presented content on those platforms. A total of 334 individuals were recruited from Amazon Mechanical Turk, with two-thirds being male (66.7%). A regression analysis showed that communal narcissism was related to increased use of Instagram and Twitter but not Reddit. Sharing content, the importance of feedback and better than average ratings had positive associations with communal narcissism. The relationship between communal narcissism and sharing on social media was fully mediated by wanting validation on social media and higher ratings of self-presented content. Communal narcissism had a notably strong relationship with wanting validation on all platforms and our results suggest that communal narcissism might be especially relevant in the context of social media use.

## 1. Introduction 

Social media use has become a routine part of daily life for a large part of the population [1]. The internet offers an array of content that can be absorbed quickly and effectively, both through the medium of text, such as obtaining information through articles and blogs [2], and through a visual format, including pictures, videos and images [3]. The introduction of such sophisticated technology to everyday life has created new norms of how people present themselves online [4], through flaunting glamourous lifestyles, flattering self-pictures and an endless supply of self-love quotes [5,6], which have formed the basis for a kind of celebrity [5] and novel professions termed “influencers” [7]. While the reasons for using social media vary [8,9], some individuals use the internet for self-enhancement and to present an idealized version of themselves. This is where personality factors such as narcissism may play a role [10], particularly because individuals high in narcissism have a previously established tendency to exaggerate desired qualities, they have unrealistically positive self-views [11,12,13], and may therefore use the internet for extensive forms of self-enhancement.

Narcissism refers to entitlement, self-absorption, self-importance, grandiose expectations of oneself and a tendency for self-enhancement [13,14,15,16,17]. The most studied subtypes of narcissism are grandiose-agentic narcissism (hereafter referred to as agentic narcissism) and vulnerable narcissism [10,16], which are subtypes of Narcissistic Personality Disorder (referred to as overt and covert narcissism) [18]. Individuals high on agentic narcissism tend to be exploitative, extroverted, attention-seeking and domineering, which is accompanied by arrogance, entitlement and high explicit self-esteem and self-enhancement. Individuals high on vulnerable narcissism share many qualities with agentic narcissism in terms of arrogance, entitlement and perceived superiority, however, vulnerable narcissists are more introverted and anxious and conceal their feelings and exploitative behaviors with deception, defensiveness, false modesty and concern for others. Individuals high on agentic narcissism generally do not concern themselves with such subtleties [13,15,16,19]. That being said, the introduction the agency–communion model of grandiose narcissism distinguished between communal and agentic self-enhancement [20], explaining how grandiosity, arrogance, entitlement and perceived superiority can also exist in a communal domain. While communal narcissism is a grandiose manifestation like agentic narcissism, it differs from agentic-grandiosity as individuals high on communal narcissism value power and grandiosity in a communal domain, by seeking admiration for being a “saint” [11,15,21,22,23]. Individuals high on communal narcissism rate themselves high on traits such as altruism, benevolence and warmth towards others [11,14], but are extremely driven by the need to validate power [24]. Their benevolent self-image does not characterize their objective communal behavior [25], and others often rate them low in actual communion [20,26]. Therefore, while individuals high on communal narcissism seek different means of acquiring power and admiration than agentic narcissists [20,21,22], factors of self-importance, unrealistically positive self-views and entitlement are shared facets of communal narcissism, vulnerable narcissism and agentic narcissism [11,20,27]. 

Unsurprisingly, these shared narcissistic tendencies relate to some online behaviors [10]. For example, individuals high on agentic narcissism use social media more frequently [28,29,30], post more pictures of themselves [31,32,33], share more information on social media [34] and engage more in addictive social media use [35]. Agentic narcissists seem to be particularly attracted to visual media [35,36,37,38]. However, less is known about narcissism and motives for using social media and preference for social media sites [11] and whether communal narcissists use social media in the same way as other narcissists. 

### The Current Study

To date, agentic narcissism and online behaviors have been the most examined, followed by vulnerable narcissism [10], but literature on communal narcissism in the online community is in its very early stages. The little available research has shown that communal narcissism relates to greater problematic behavior offline, such as peer-perceived aggression [26], counterproductive workplace behavior, which often relates to interpersonal conflict [39], and communal narcissistic statements on Facebook, which are generally viewed negatively by others [40]. Therefore, as agentic narcissism has been found related to various aspects of social media use [10,30,37], and considering similarities in grandiosity, motives and manifestation [11,12,13,20,21,22,25,40], it warrants examination of how communal narcissism as a construct relates to social media use and behavior, especially given previously established problematic behaviors. Currently, to our knowledge, no research has examined communal narcissism in the online community, controlling for agentic narcissism and vulnerable narcissism. This is important because even though communal narcissism shares some characteristics with other types of narcissism, it is a distinct concept. 

Therefore, as communal narcissism has been severely neglected from the literature on narcissistic tendencies and social media to date, it remains unknown if communal narcissistic behaviors and qualities apply in the online community as well. It is expected that communal narcissism will display a prominent relationship with social media use, as with other manifestations of narcissism [10], due to shared facets of power-seeking, entitlement and self-enhancement [11,15,25]. Previous research has demonstrated that narcissism correlates positively with social media usage [28,29,30], relates to increased sharing of information on both visual and text based-social media platforms [34] and has been linked to greater usage of the social networking sites Twitter (a mixed platform) [37] and Instagram (a visual platform) [36]. Therefore, a similar pattern is expected for communal narcissism and social media use. However, these relationships seem to differ on the nature of the representation of content on the sites. Notably, a relationship between narcissism and problematic internet use has been found to be mediated by visual social media, a relationship not found for text-based social media [38], and posting self-pictures on visual social media relates to higher levels of narcissism [30,31,32]. Therefore, given the prior established relationships between agentic narcissism and visual social media, it was hypothesized that communal narcissism would have a stronger relationship with use and sharing on visual social media, indicating that communal narcissism relates similarly to social media platforms as the agentic type of narcissism.

The focus of previous social media and narcissism studies has also largely been on Facebook use [10,34,40,41,42,43,44]. A meta-analysis on agentic and vulnerable narcissism and social media reported that previous literatures’ significant limitations included a lack of understanding of narcissism and other social media sites (e.g., Instagram and Reddit) and other social networking mechanisms [10]. It was therefore decided to include a text-based, visual or mixed distinction in the current study, which also coincided with previous findings with different results due to text/visual content representation [38]. Furthermore, individuals high on narcissism tend to seek more feedback on their social media posts and partake in excessive forms of self-promotion [35,37,39]. Thus, it was hypothesized that this excessive need for admiration will be present for communal narcissists in the online community as well in the form of wanting electronic feedback. Lastly, because communal narcissism is generally related to unrealistically positive self-views [11,14,23], it was also expected that individuals high on communal narcissism would rate their own self-presented content online as above average.

The goal of this study was to examine the relationships between social media use and communal narcissism and assess the use of popular social media sites, which possessed visual, text-based and mixed content representation, to address previous gaps in the literature [10]. First, Instagram use was examined [3]. Instagram is a popular visual social media platform that allows sharing of images and videos, where users can receive “likes” and “comments” on their posts [45]. Secondly, Reddit use was examined. Reddit is a primarily a text-based site, where users can ask questions, share news, scientific research, opinions and theories and even share intimate information and rank other users’ posts using “karma-points” [46,47]. Lastly, Twitter use was examined, as it portrays a combination of text-based and visual posts [3]. 

Previous studies have explored narcissism and frequency of sharing [34,48] and frequency of use [28,29] which was included in the current analysis, with the addition of rating one’s own content and rating the importance of receiving feedback on social media. Some motives for social media, such as wanting admiration and wanting followers, have been found to have a strong relationship with narcissism [37]. Given that vulnerable, agentic [49] and communal narcissists [11] seek validation, we examined this motive for sharing behavior for individuals high on communal narcissism. In line with the previous findings [20,50,51] and given the previously established relationships between communal narcissism and overclaiming [20] and a desire for praise for communal behavior [11], it was expected that communal narcissism would be positively associated with certain motives for social media use, and that these motives could ultimately mediate the relationship between narcissism and actual sharing and use of social media. Notably, rating self-presented content as superior to others and high importance of receiving feedback on social media were examined as possible motives for frequency of sharing on social networking sites for individuals high on communal narcissism [52]. Furthermore, as demographic information relates to narcissism, with elevated levels among younger people [53] and men [54], they were included as covariates. 

**Figure 1:**
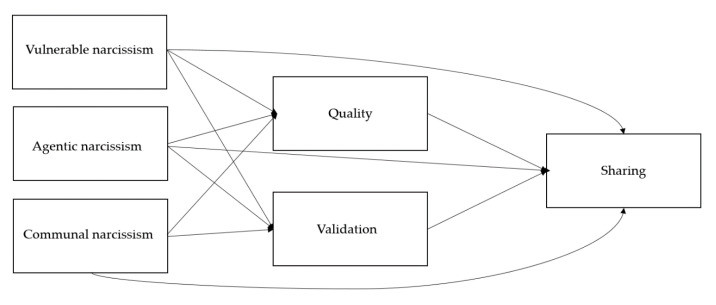
Hypothesized model of relationships between narcissism and social media behaviors and motives.

To demonstrate, Figure 1 shows the theoretical model between different types of narcissism and social media behaviors. We proposed that narcissism would relate to sharing content on social media, and that this relationship would be mediated by motives for social media use. In this way, narcissists may share information on social media because they believe that their content is of greater quality than content from other people, because of their inflated sense of self-worth and unrealistically positive self-views [11,14,23]. Narcissists could also use social media as a way of seeking out validation and admiration from other people, which could be more prominent among vulnerable and communal narcissists because they tend to be concerned about the opinions of other people more than agentic narcissists.

## 2. Method

### 2.1. Participants and Procedure

To find out the required sample size, a priori power analysis was conducted. Based on previous research [28,29,30], a probable effect size when assessing the association between narcissism and frequency of social media use was thought to be medium sized. To detect a similar effect (*f^2^* = 0.15, with statistical power (1 − β) = 0.95, and five predictors), a sample size of N = 138 was required [55,56]. 

Participants in this study were 334 in total, with 66.7% male and 32.3% female. The age of the sample ranged from 18 to 74, with most participants reporting being aged 25 to 34 years old (48.8%). Most participants came from North America (57.5%), then Asia (19.5%), followed by Europe (9.9%), Latin America and the Caribbean (7.8%) and Middle East and Africa (5.1%). Originally, a total of 360 participants were recruited for the study, but 26 participants failed the quality control test of answering too quickly and thus were excluded, as established in previous studies [57,58] to ensure valid and reliable answers. Participants were recruited through Amazon Mechanical Turk, where they were paid $1 for their participation. Amazon Mechanical Turk has been found to be effective for data collecting, and as reliable as other methods [59], which is especially effective when the research is not extremely time-consuming, nor requires immense concentration [60]. Amazon Mechanical Turk participants represent a more diverse sample than various other methods, e.g., undergraduate students [59]. It was thereby deemed appropriate for the current study. The only inclusion criterion for participants was to have reached the consenting age of 18, which was an important criterion as the goal was to include a diverse sample to increase the generalizability of the results. All participants were informed of what was expected of them and were asked to give their consent before starting the survey by signing an electronic consent form. The survey followed the APA ethical principles and code of conduct and was carried out in accordance with the principles of the Declaration of Helsinki.

### 2.2. Measures 

Demographic information. Participants were asked to indicate where they came from, their gender (male or female) and age, where the response options for age were 18–24, 25–34, 35–44, 45–54, 55–64, 65–74, 75–84 and 85 or older. 

Social media behavior. Participants were asked whether they used Reddit, Instagram or Twitter separately, and if the answer was yes (coded 1) compared to no (coded 0), more questions were displayed regarding their social media behavior on those sites. If the answer was no to use, they skipped the following question regarding that specific platform. If yes, participants were asked how frequently the platform was used. Secondly, how often they shared opinions (Reddit), self-pictures (Instagram) or images and posts (Twitter). The answer choices for the first two questions ranged from 1 (*very often*) to 5 (*never*). Third, to determine need for validation, participants were asked how important it was for them to earn karma-points (Reddit), likes (Instagram) or likes/retweets (Twitter) on their posts, where the answers ranged from 1 (*extremely important*) to 5 (*not at all important*). Lastly, participants were asked to rate the quality of their own social media content, where the answers were: *far below average, moderately below average, slightly below average, average, slightly above average, moderately above average* and *far above average*. Including the possibility to state “*I do not share connect on Reddit/Instagram/Twitter*”. 

Narcissism. To measure communal narcissism, the Communal Narcissism Inventory (CNI) was used [20,61]. The CNI is a 16-item scale designed to measure narcissism in a communal domain and contains statements such as “*I am the most helpful person I know*” and “*I am the best friend someone can have*”. Participants rate each item on a scale 1 (*strongly agree*) to 7 (*strongly disagree*). The internal consistency in the current sample was good (α = 0.96). 

To measure agentic narcissism and control for its effects, the Narcissistic Personality Inventory 13-item scale (NPI−13) was used [62] which is a forced answer scale where participants are asked to choose between a (A) narcissistic or (B) non-narcissistic statement, such as “*I insist upon getting the respect that is due to me*” or “*I usually get the respect I deserve*” [63]. The internal consistency in the current sample was acceptable (α = 0.72). 

To measure vulnerable narcissism, the Hypersensitive Narcissism scale (HSNS) was used. The HSNS is a 10-items scale that includes statements such as: “*I dislike sharing credit of an achievement with others*” where answers ranged from 1 (*very characteristic*) to 5 (*very uncharacteristic*) [64,65]. The internal consistency in the current sample was good (α = 0.83). While the CNI differs from measures of agentic narcissism [20], narcissistic subtypes have shared factors, motives and tendencies [22,23,27]. Therefore, it was decided to control for HSNS and NPI−13 in the data analysis to properly determine the effects CNI had directly on the social media variables.

### 2.3. Method of Analysis

To analyze social media use, Pearson correlation coefficients were calculated [66] between the Communal Narcissism Scale and each social media platform and behavior, in line with past research on narcissism and social media [10,29,33,34,42,44]. Furthermore, to understand the association between narcissism and individual behavior, hierarchical logistic and linear regressions were performed, with gender and age entered as covariates and to understand the effect of communal narcissism independently by controlling for agentic and vulnerable narcissism in the second step of the analysis. Lastly, the hypothesized motives of wanting validation and believing one’s own content to be higher quality were entered into a mediation analysis to understand the mediation effects between communal narcissism and sharing on social media.

The statistical package for social sciences (SPSS) was used for the analysis [67]. Prior to the statistical analysis, the data set was examined for any breaches in assumptions. Given the size of the samples, normality could be assumed, which was consistent with the Q-Q plots examined. No large violations in assumptions were recorded, therefore the data were deemed suitable for parametric testing. 

To test whether motives of using social media (quality and validation) mediated the relationship between narcissism and sharing on social media, we carried out a structural equation path model. The path model was tested using Mplus, version 6.12 [68] using 5000 bootstrapped samples with good model fit [69]. All reported path model coefficients are standardized values.

## 3. Results

A total of 87, 81, and 76% of participants responded as using Reddit, Instagram and Twitter, respectively (see Table 1). The correlation between communal and agentic narcissism was *r_p_* = 0.497, *p* < 0.01; between communal and vulnerable narcissism *r_p_* = 0.557, *p* < 0.01 and between communal and vulnerable narcissism *r_p_* = 0.480, *p* < 0.01. To explore the relationship between narcissism and social media use, Pearson correlation coefficients were examined. The results from the analysis are presented in Table 1, which includes correlation coefficients between communal, agentic, and vulnerable narcissism and Reddit, Instagram and Twitter usage. 

As Table 1 shows, communal narcissism had a medium to strong positive relationship with sharing behavior, importance of feedback and rating quality of their own posts for Reddit, Instagram and Twitter, similar to agentic and vulnerable narcissism. Additionally, communal narcissism was positively associated with use and frequency of use of Instagram and Twitter just like agentic and vulnerable narcissism. However, communal and agentic narcissism did not correlate with use and frequency of use for Reddit as vulnerable narcissism did.

To test whether communal narcissism predicted social media behaviors, in addition to agentic and vulnerable narcissism, we ran a logistic regression analysis (see Table 2) and hierarchical linear regression analyses (see Table 3, Table 4, Table 5 and Table 6). We entered the independent variables simultaneously after ascertaining that no assumptions were violated, including the assumption of multicollinearity (tolerance scores were higher than 0.6 and VIF scores were below 1.7). In the first step, we entered gender and age as independent variables and in the second step, we added agentic and vulnerable narcissism as independent variables. Finally, in the third step, communal narcissism was added as an independent variable. 

When controlling for demographics factors and agentic and vulnerable narcissism, communal narcissism was positively related to all the social media behaviors, except for Reddit use and frequency of use (see Table 2, Table 3, Table 4, Table 5 and Table 6). This suggests that communal narcissism can predict using Instagram and Twitter, sharing on all platforms, wanting feedback and higher ratings of self-presented content even when controlling for agentic and vulnerable narcissism.

**Figure 2:**
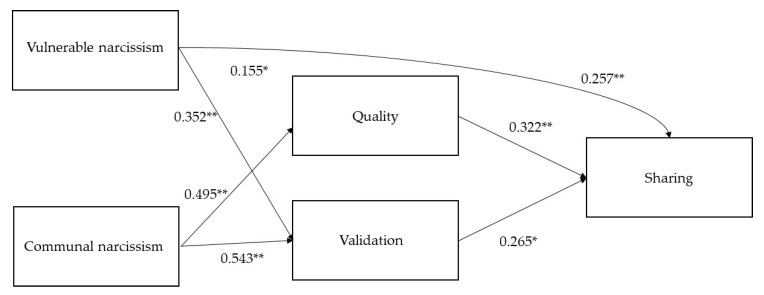
Final model of the relationship between narcissism and social media behaviors and motives. * Significant at the 5% level; ** significant at the 1% level.

To understand the mediating effects, all three narcissism variables, the motives and sharing were entered into a mediation analysis. Figure 2 shows the final mediation model between narcissism and social media behaviors. The model has been adapted to reflect that agentic narcissism did not relate to social media motives (quality and validation) or social media sharing. Communal narcissism relates strongly both to believing that one’s content is of superior quality as well as seeking validation. Vulnerable narcissism also has positive relationships with validation and quality, although weaker for the latter. Both quality and validation relate to greater sharing.

Significant indirect effects emerged from communal narcissism to sharing through both validation (z = 0.144, *p* = 0.007) and quality (z = 0.159, *p* = 0.010). These social media motivators fully mediated the relationship between communal narcissism and sharing (direct effect = 0.141, *p* = 0.135). In addition, there was a significant indirect effect from vulnerable narcissism to sharing through validation (z = 0.093, *p* = 0.015), which partially mediated the relationship between vulnerable narcissism and sharing (direct effect = 0.257, *p* < 0.001). Narcissism explains a large amount of variance in validation (r^2^ = 0.631) and less variance in quality (r^2^ = 0.355). Together, narcissism and motives also explain variation in sharing well (r^2^ = 0.670).

## 4. Discussion

This study analyzed communal narcissism in relation to social media behavior and motives, with the intention of adding to the literature on communal narcissism within the online community. While agentic narcissism has been extensively covered in relation to online use, with vulnerable narcissism covered to some extent [10], communal narcissism has been largely missing from the online literature to date. As the results indicate, communal narcissism had a positive relationship with use of Instagram and Twitter, frequency of sharing on all platforms, importance of receiving feedback on all platforms and a higher quality-rating of self-presented content on all platforms. Similar to previous findings, narcissism correlated with frequency of sharing, validation and quality-rating [10,29,31,34], with communal narcissism maintaining its unique association with the social media behaviors when controlling for agentic and vulnerable narcissism. This implies the importance of communal narcissism when studying narcissism within social media [11].

Individuals high on communal narcissism seemed at least as likely to use Instagram as individuals high on agentic narcissism, which might be comparable to previous studies that have found that agentic narcissists relate more strongly to visual social media [34,38]. In general, it might be the case that the use of visual social media content appeals more to narcissistic individuals, as some studies have indicated [34,38]. In fact, in our study we saw a lack of association between communal narcissism and using Reddit, a primarily text-based platform [46,47]. 

All things considered, communal narcissism is related to higher prosocial self-enhancement and is inherently rooted in communion [25]. Therefore, given these findings, the distinct communal narcissistic traits of overclaiming [20] and a desire for appraisal for communal behavior [11] may drive these behaviors and underlying motives as the mediation analysis supported. Thus, social media can serve as means for attention and validation from others through the internet and yield desired feelings of grandiosity, entitlement and feelings of superiority (i.e., through better than average ratings), from behind a screen. However, further research is needed to confirm these ideas.

However, the three platforms examined possess different kinds of use, not solely related to visual or text-based representations. For example, Twitter is recognized for online activism [70,71] and Instagram is sometimes used for financial reasons [7], which might affect use and initiatives, unrelated to the current focus of comparing visual and text-based media. Future studies need to take this into account by adding predictors to their models. Further limitations of the study must be noted. First, the focus was on believed narcissistic use of those platforms e.g., sharing opinions and sharing self-pictures, which might also have affected responses regarding sharing tendencies. Secondly, self-reported questionnaires rely on the ability and willingness of participants to give accurate data about themselves. For example, people may have difficulty providing accurate data about how much social media they use, especially when differentiated by platform. This issue is further exacerbated when studying narcissists, who may distort their answers to self-report questionnaires because of their established tendency to enhance their own performance [72]. Future studies should include measures to counteract this problem, such as a social desirability scale. Third, this study examined correlation, not causation, therefore we cannot conclude whether the social media behaviors and preferences are increased by narcissism or vice versa, as previously suggested [38]. Therefore, further research is needed to understand the nature of these relationships. In addition, age was assessed using categorical age ranges, rather than continuously, which may have impacted the results by removing variability. Therefore, future studies should investigate the role of background variables, such as age, nationality, gender and education, and how these might shape the relationships between narcissism and social media use. An important component of investigating these would be for recruitment to specifically target subgroups that allow for comparison across the demographic dimensions. Furthermore, the relationships investigated here are likely to be complex and bi-directional. For example, a feedback loop from sharing content to both quality and validation could be expected, with greater sharing increasing the amount of validation and feedback received from others, which in, in turn, could then lead to greater sharing. Given the cross-sectional nature of the current data, fully investigating the temporality of these relationships is not feasible, but future studies should aim to further this theoretical framing and endeavor to understand the causation and directional nature of the model.

## 5. Conclusions

That being said, this study presented new findings regarding communal narcissism in the online community. Interestingly, as agentic narcissism has been mostly covered in the literature, these results indicated that communal narcissism displays strong relationships with social media use and specific behaviors as well, and motives for doing so. In addition, while displaying a preference for the visual platform Instagram, upon choosing another social networking site, sharing content, wanting validation and quality-ratings were just as prominent for text-based sites. Perhaps a visual format has a stronger appeal to narcissism, but narcissism does relate to certain behaviors upon choosing any platform, which is an interesting aspect for future studies. Furthermore, this study underlines the importance of properly separating the effects that different manifestations of narcissism can have on various behaviors, both online and in direct communication. More research in needed on communal narcissism in relation to social media use and other online behaviors. In addition, more research is needed to understand causal relationships of narcissism and social media use and a proper separation of different genders, age groups and cultures to generalize the overall effects.

## Figures and Tables

**Table 1:** Means, standard deviations (SD) and Pearson correlation coefficients for the three types of narcissism (communal, agentic and vulnerable) and social media behavior.

	N	Mean (SD)	Communal Narcissism	Agentic Narcissism	Vulnerable Narcissism
Reddit (*text-based*)					
Use	334	0.865 (0.342)	−0.053	−0.092	−0.124 *
Frequency of use	289	2.291 (0.982)	0.100	0.045	0.309 **
Frequency of sharing opinions	282	2.734 (1.121)	0.528 **	0.410 **	0.525 **
Importance of feedback	285	2.940 (1.366)	0.683 **	0.479 **	0.570 **
Rating quality	289	3.353 (1.706)	0.435 **	0.286 **	0.349 **
Instagram (*visual*)					
Use	334	0.811 (0.392)	−0.376 **	−0.277 **	−0.268 **
Frequency of use	271	1.934 (0.896)	0.354 **	0.135 *	0.331 **
Frequency of sharing self-portraits	271	2.520 (1.091)	0.516 **	0.389 **	0.512 **
Importance of feedback	269	2.639 (1.200)	0.532 **	0.432 **	0.515 **
Rating quality	271	3.122 (1.499)	0.386 **	0.184 **	0.347 **
Twitter (*visual and text-based*)					
Use	333	0.757 (0.430)	−0.251 **	−0.167 **	−0.106
Frequency of use	253	2.071 (0.969)	0.272 **	0.069	0.336 **
Frequency of sharing opinions	249	2.606 (1.146)	0.512 **	0.304 **	0.463
Frequency of sharing pictures	253	2.791 (1.208)	0.580 **	0.409 **	0.598 **
Importance of feedback	251	2.637 (1.290)	0.637 **	0.447 **	0.635 **
Rating quality	253	3.126 (1.548)	0.423 **	0.124	0.286 **

* Significant at the 5% level; ** significant at the 1% level.

**Table 2:** Logistic regression predicting social media usage for Reddit, Instagram and Twitter, showing unstandardized coefficients (b), standard errors (SE) and the odds ratios for the unstandardized coefficients (Exp(b)).

	Reddit			Instagram			Twitter		
	b	SE	Exp(b)	b	SE	Exp(b)	B	SE	Exp(b)
Step 1									
Males	0.241	0.342	1.273	−1.048 **	0.376	0.351	0.142	0.276	1.153
Age	−0.103	0.144	0.902	−0.373 **	0.128	0.688	−0.038	0.120	0.963
Nagelkerke R^2^	0.006			0.086 **			0.002		
Step 2									
Males	0.248	0.345	1.282	−1.175 **	0.398	0.309	0.124	0.280	1.132
Age	−0.058	0.145	0.943	−0.317 *	0.136	0.728	−0.022	0.121	0.978
AN	−0.035	0.062	0.966	−0.173 **	0.059	0.841	−0.117 *	0.050	0.889
VN	−0.041	0.027	0.960	−0.053 *	0.026	0.949	−0.006	0.022	0.994
Nagelkerke R^2^	0.033			0.217			0.042 ^†^		
Step 3									
Males	0.250	0.345	1.284	−1.260 **	0.412	0.284	0.114	0.288	1.120
Age	−0.071	0.147	0.931	−0.243 ^†^	0.142	0.784	0.034	0.125	1.035
AN	−0.048	0.067	0.953	−0.072	0.064	0.930	−0.042	0.053	0.959
VN	−0.045	0.029	0.956	−0.027	0.029	0.973	0.026	0.025	1.027
CN	0.006	0.010	1.006	−0.038 **	0.010	0.963	−0.032 **	0.009	0.968
Nagelkerke R^2^	0.034			0.285			0.107 **		

^†^*p* < 0.10, * *p* < 0.05, ** *p* < 0.01 (two-tailed). AN = agentic narcissism, VN = vulnerable narcissism, CN = communal narcissism.

**Table 3:** Multiple linear regression predicting frequency of social media use for Reddit, Instagram and Twitter, showing unstandardized coefficients (b), standard errors (SE) and standardized coefficients (Beta).

	Reddit			Instagram			Twitter		
	b	SE	Beta	b	SE	Beta	b	SE	Beta
Step 1									
Males	−0.065	0.126	−0.031	−0.099	0.117	−0.053	0.068	0.137	0.032
Age	0.030	0.057	0.032	0.086	0.055	0.097	0.080	0.060	0.086
R^2^	0.002			0.012			0.008		
Step 2									
Males	−0.093	0.120	−0.045	−0.065	0.112	−0.035	0.054	0.130	0.026
Age	0.013	0.054	0.014	0.080	0.053	0.091	0.037	0.058	0.039
AN	−0.045 *	0.021	−0.140	0.007	0.021	0.022	−0.034	0.023	−0.101
VN	0.053 **	0.009	0.384	0.040 **	0.009	0.299	0.048 **	0.009	0.362
R^2^	0.117 **			0.107 **			0.113 **		
Step 3									
Males	−0.091	0.12	−0.044	−0.034	0.110	−0.018	0.050	0.130	0.024
Age	0.016	0.054	0.018	0.078	0.052	0.088	0.024	0.058	0.026
AN	−0.037	0.023	−0.116	−0.010	0.021	−0.032	−0.045	0.024	−0.134
VN	0.056 **	0.010	0.407	0.027 **	0.009	0.198	0.041 **	0.010	0.303
CN	−0.003	0.004	−0.067	0.013 **	0.009	0.243	0.007 ^†^	0.004	0.134
R^2^	0.120 **			0.148 **			0.124 **		

^†^*p* < 0.10, * *p* < 0.05, ** *p* < 0.01 (two-tailed). AN = agentic narcissism, VN = vulnerable narcissism, CN = communal narcissism.

**Table 4:** Multiple linear regression predicting frequency of sharing of social media for Reddit, Instagram and Twitter, showing unstandardized coefficients (b), standard errors (SE) and standardized coefficients (Beta).

	Reddit			Instagram			Twitter Opinions			Twitter Pictures		
	b	SE	Beta	b	SE	Beta	b	SE	Beta	b	SE	Beta
Step 1												
Males	0.132	0.146	0.056	−0.167	0.142	−0.073	−0.232	0.158	−0.096	0.021	0.167	0.008
Age	−0.035	0.066	−0.032	0.105	0.067	0.098	0.065	0.070	0.061	0.140 ^†^	0.074	0.123
R^2^	0.004			0.015			0.013			0.015		
Step 2												
Males	0.151	0.121	0.064	−0.053	0.119	−0.023	−0.206	0.142	−0.085	0.056	0.135	0.022
Age	−0.048	0.055	−0.045	0.110 ^†^	0.056	0.102	−0.010	0.063	−0.009	0.047	0.060	0.041
AN	0.080 **	0.022	0.215	0.090 **	0.022	0.229	0.043 ^†^	0.025	0.113	0.072 **	0.024	0.176
VN	0.068 **	0.009	0.426	0.070 **	0.009	0.428	0.060 **	0.010	0.393	0.081 **	0.010	0.496
R^2^	0.317 **			0.323 **			0.217 **			0.366 **		
Step 3												
Males	0.150	0.115	0.063	−0.001	0.113	−0.001	−0.222	0.135	−0.092	0.042	0.128	0.016
Age	−0.066	0.052	−0.062	0.105 *	0.053	0.098	−0.046	0.061	−0.043	0.010	0.057	0.009
AN	0.035	0.022	0.095	0.061 **	0.022	0.155	0.011	0.025	0.028	0.040 ^†^	0.024	0.098
VN	0.051 **	0.009	0.319	0.047 **	0.010	0.287	0.037 **	0.011	0.241	0.058 **	0.010	0.354
CN	0.019 **	0.003	0.330	0.021 **	0.004	0.335	0.021 **	0.004	0.348	0.021 **	0.004	0.323
R^2^	0.387 **			0.401 **			0.292 **			0.431 **		

^†^*p* < 0.10, * *p* < 0.05, ** *p* < 0.01 (two-tailed). AN = agentic narcissism, VN = vulnerable narcissism, CN = communal narcissism.

**Table 5:** Multiple linear regression predicting feedback/validation of social media for Reddit, Instagram and Twitter, showing unstandardized coefficients (b), standard errors (SE) and standardized coefficients (Beta).

	Reddit			Instagram			Twitter		
	b	SE	Beta	b	SE	Beta	b	SE	Beta
Step 1									
Males	−0.010	0.180	−0.003	−0.175	0.158	−0.069	−0.114	0.179	−0.041
Age	0.016	0.081	0.012	0.085	0.075	0.071	0.151 ^†^	0.079	0.123
R^2^	0.000			0.010			0.017		
Step 2									
Males	0.018	0.143	0.006	−0.039	0.131	−0.015	−0.075	0.138	−0.027
Age	−0.004	0.064	−0.003	0.095	0.062	0.080	0.042	0.062	0.034
AN	0.122 **	0.025	0.270	0.122 **	0.024	0.282	0.085 **	0.025	0.192
VN	0.084 **	0.011	0.438	0.072 **	0.010	0.399	0.094 **	0.010	0.533
R^2^	0.377 **			0.332 **			0.427 **		
Step 3									
Males	0.010	0.121	0.003	0.021	0.124	0.008	−0.094	0.127	−0.034
Age	−0.037	0.054	−0.028	0.090	0.058	0.076	−0.006	0.057	−0.005
AN	0.034	0.023	0.075	0.090 **	0.024	0.208	0.045 ^†^	0.024	0.101
VN	0.050 **	0.010	0.261	0.047 **	0.011	0.261	0.065 **	0.010	0.367
CN	0.036 **	0.004	0.530	0.023 **	0.004	0.330	0.027 **	0.004	0.380
R^2^	0.555 **			0.407 **			0.517 **		

^†^*p* < 0.10, ** *p* < 0.01 (two-tailed). AN = agentic narcissism, VN = vulnerable narcissism, CN = communal narcissism.

**Table 6:** Multiple linear regression predicting quality rating of social media for Reddit, Instagram and Twitter, showing unstandardized coefficients (b), standard errors (SE) and standardized coefficients (Beta).

	Reddit			Instagram			Twitter		
	b	SE	Beta	b	SE	Beta	b	SE	Beta
Step 1									
Males	0.087	0.222	0.024	0.043	0.197	0.014	−0.094	0.214	−0.028
Age	0.092	0.100	0.056	0.074	0.093	0.050	0.165 ^†^	0.094	0.113
R^2^	0.004			0.003			0.014		
Step 2									
Males	0.112	0.208	0.031	0.120	0.187	0.038	−0.093	0.208	−0.028
Age	0.079	0.094	0.048	0.070	0.088	0.047	0.111	0.093	0.076
AN	0.097 **	0.037	0.171	0.041	0.035	0.076	−0.001	0.038	−0.003
VN	0.059 **	0.016	0.246	0.068 **	0.014	0.301	0.054 **	0.015	0.255
R^2^	0.134 **			0.116 **			0.077 **		
Step 3									
Males	0.098	0.201	0.027	0.182	0.182	0.058	−0.114	0.198	−0.035
Age	0.053	0.091	0.032	0.064	0.086	0.043	0.054	0.089	0.037
AN	0.033	0.038	0.058	0.007	0.035	0.013	−0.050	0.089	−0.095
VN	0.034 *	0.016	0.139	0.041 **	0.015	0.182	0.018	0.037	0.087
CN	0.027 **	0.006	0.315	0.025 **	0.006	0.286	0.032 **	0.016	0.384
R^2^	0.197 **			0.173 **			0.169 **		

^†^*p* < 0.10, * *p* < 0.05, ** *p* < 0.01 (two-tailed). AN = agentic narcissism, VN = vulnerable narcissism, CN = communal narcissism.

## Data Availability

The data presented in this study are available on request from the corresponding author. The data are not publicly available due to ethical restrictions.
